# Mind Causality: A Computational Neuroscience Approach

**DOI:** 10.3389/fncom.2021.706505

**Published:** 2021-07-08

**Authors:** Edmund T. Rolls

**Affiliations:** ^1^Oxford Centre for Computational Neuroscience, Oxford, United Kingdom; ^2^Department of Computer Science, University of Warwick, Coventry, United Kingdom

**Keywords:** the mind-brain problem, causality, neuronal networks, neural computation, consciousness, computational neuroscience, dualism, supervenience

## Abstract

A neuroscience-based approach has recently been proposed for the relation between the mind and the brain. The proposal is that events at the sub-neuronal, neuronal, and neuronal network levels take place simultaneously to perform a computation that can be described at a high level as a mental state, with content about the world. It is argued that as the processes at the different levels of explanation take place at the same time, they are linked by a non-causal supervenient relationship: causality can best be described in brains as operating within but not between levels. This mind-brain theory allows mental events to be different in kind from the mechanistic events that underlie them; but does not lead one to argue that mental events cause brain events, or vice versa: they are different levels of explanation of the operation of the computational system. Here, some implications are developed. It is proposed that causality, at least as it applies to the brain, should satisfy three conditions. First, interventionist tests for causality must be satisfied. Second, the causally related events should be at the same level of explanation. Third, a temporal order condition must be satisfied, with a suitable time scale in the order of 10 ms (to exclude application to quantum physics; and a cause cannot follow an effect). Next, although it may be useful for different purposes to describe causality involving the mind and brain at the mental level, or at the brain level, it is argued that the brain level may sometimes be more accurate, for sometimes causal accounts at the mental level may arise from confabulation by the mentalee, whereas understanding exactly what computations have occurred in the brain that result in a choice or action will provide the correct causal account for why a choice or action was made. Next, it is argued that possible cases of “downward causation” can be accounted for by a within-levels-of-explanation account of causality. This computational neuroscience approach provides an opportunity to proceed beyond Cartesian dualism and physical reductionism in considering the relations between the mind and the brain.

## Introduction

A neuroscience-based approach has recently been proposed for the relation between the mind and the brain (Rolls, 2021a). The proposal is that events at the sub-neuronal, neuronal, and neuronal network levels take place simultaneously to perform a computation that can be described at a high level as a mental state, with content about the world. It is argued that as the processes at the different levels of explanation take place at the same time, they are linked by a non-causal supervenient relationship: causality can best be described in brains as operating within but not between levels. This mind-brain theory allows mental events to be different in kind from the mechanistic events that underlie them; but does not lead one to argue that mental events cause brain events, or vice versa: they are different levels of explanation of the operation of the computational system. This approach may provide a way of thinking about brains and minds that is different from dualism and from reductive physicalism (Kim, 2011), and which is rooted in the computational processes that are fundamental to understanding brain and mental events, and that mean that the mental and mechanistic levels are linked by the computational process being performed. Explanations at the different levels of operation may be useful in different ways (cf Dennett, 1991). For example, if we wish to understand how arithmetic is performed in the brain, description at the mental level of the algorithm being computed will be useful. But if the brain operates to result in mental disorders, then understanding the mechanism at the neural processing level may be more useful, in for example the treatment of psychiatric disorders.

In terms of levels of explanation that apply to the brain and mental operations, a number of different levels of explanation can be identified (Rolls, 2021a). They include ion channels in neurons influenced by neurotransmitters released at the tens of thousands of synapses on each neuron through which currents pass to influence the firing rate of individual neurons; neuronal biophysics that influences how these currents are converted into firing rates; the firing rates of individual neurons; the computations performed by populations of neurons often involving collective computations as in attractor networks and competitive networks; how the activity of populations of neurons is reflected by functional neuroimaging; to behavioral and cognitive effects, including mental operations, verbal reports, and phenomenal consciousness (Rolls, 2016, 2020, 2021c,a). I regard these as different levels of explanation of the operation of a computational system such as the brain.

Some key points are developed further here. One is what the implications are for theories of causality. A second key point is which level of explanation may provide a more accurate account for the cause of a choice or action: the mental level, or the computational neuroscience level. A third key point is whether there are any cases in which it might be appropriate to provide a “downward causation” account, in which a higher level of the system causes effects at a lower level.

## Causality

### Intervention

The most widely considered approach to causality is an interventionist account (Woodward, 2005, 2015, 2020, 2021b; Craver and Bechtel, 2007; Kim, 2011). If one intervenes to remove a potential cause, and the putative effect no longer occurs, then that makes it more likely that the potential cause does cause the putative effect. [More formally, where X and Y are variables, X causes Y if there are some possible interventions that would change the value of X and if such intervention were to occur, a regular change in the value of Y would occur (Woodward, 2020, 2021b)]. So this is a necessary condition for causality. But I now argue that it is not a sufficient condition, at least in relation to mental and brain events.

### Causality Operates Within a Level of Operation and Explanation, Not Between Levels

The argument follows from my approach to causality in minds and brains, that causality can best be considered as operating within a level of explanation, and not between levels. So a second condition I argue that needs to be satisfied for causality is that the cause and effect are within the same level of explanation. I made it relatively clear in my earlier exposition (Rolls, 2021a) that level here might refer to the mental level, for example a cause provided verbally by an individual for an action; or it might be at a computational level for what might be computed by a population of neurons; or it might be at the single neuron level; or it might be at the level of transmitters influencing ion channels to make neurons fire more or less, etc. (Rolls, 2021a). The bases for this argument, that causality operates within but not between the levels of operation and explanation of the system are set out for both minds / brains and for computers by Rolls (2021a). The bases include the point that the processes that occur at the different levels can occur simultaneously (for example the mental and brain event, or the mathematical or logic operation performed by a computer and the current flow within its arithmetic logic unit), whereas causal processes can be understood to involve sequences of events in time with the operations performed within a level.

This point is important. If all I held was an interventionist account of causality, then I might find the conditions satisfied that a mental event might cause a brain event, and it would be difficult to exclude that in terms of possible interventions. But that would be incorrect, if one holds that causality should best be considered to operate within a level of explanation, and not between levels of explanation, as set out elsewhere (Rolls, 2021a).

In brief, an interventionist account might not be able to reject the hypothesis that mental events cause brain events, for particular mental events will always and indissolubly be associated with brain events. The reason for this is that an interventionist account of causality might diagnose cases of causality that act across levels of explanation. The implication is that the interventionist account alone will not suffice as a criterion for causality, at least for operations in brains and computers. The criteria would have to include also a restriction to events at the same level of explanation.

### Temporal Order

Temporal order may also be useful as a condition for whether causality applies. At its simplest, a cause cannot follow an effect, as least in the macro world that is considered here. In neuroscience (and this may be different from quantum physics), we think that when causes produce effects a time delay is a useful indicator. Following this thinking, when one step of a process at one level of explanation moves to the next step in time, we can speak of causality that would meet the criteria for Granger causality where one time series, including the time series being considered, can be used to predict what happens at the next step in time (Granger, 1969; Bressler and Seth, 2011; Ge et al., 2012). In relation to neuroscience, the timing of a set of events measured with an accuracy of in the order of 10 ms and for a sufficient period on either side of the causal event being tested would suffice. This time scale, with very many time-steps of 10 ms on each side of the putative cause-effect relationship should be adequate, in that the time-scale of computation in the brain is in the order of 10–15 ms, which is the time that it might take a pattern association network, a competitive network, or even an attractor network to perform its computation (Rolls, 2021c) (see below).

The implication of temporal order for levels of explanation and causality is that when we consider the relationship between processes described at different levels of explanation, such as the relation between a step in the hardware in a computer and a step in the software, then these processes may occur simultaneously, and be inextricably linked with each other, and just be different ways of describing the same process, so that temporal (Granger) causality does not apply to this relation between levels, but only within levels. The whole processing can then be specified from the mechanistic level of neuronal firings, etc., up through the computational level to the cognitive and behavioral level, as described elsewhere (Rolls, 2021a,c). The thrust of this argument is that temporal order is also a useful criterion to identify causality, at least at the macro level of events in the mind and the brain; and in computers.

### Criteria for Causality

These points lead to my proposal for conditions that need to be tested for and satisfied to assess whether causality applies in a particular case, as follows:

1. Interventionist tests need to be satisfied. Interventionist tests provide conditions that need to be satisfied for causality, but they are not sufficient conditions for causality to be identified.
2. The events should be at the same level of explanation. Further details are described elsewhere (Rolls, 2021a).
3. Temporal order needs to be satisfied, as set out above. Details about how this applies in the brain are provided elsewhere (Rolls, 2021a).

Criterion (1), interventionism, follows Woodward (2005, 2015), and is what I would describe as a way of testing whether causality can be excluded in a particular case, rather than a substantive account of causality.

Criterion (2), that causality operates within but not between levels of explanation, moves beyond a purely interventionist account of causality, and is a proposal that I made, and elaborated in considering how causality operates within a multilevel system such as the mind and brain, and the software and hardware of a computer (Rolls, 2021a).

Criterion (3), temporal order, also goes beyond a purely interventionist account, and is helpful partly because it helps to diagnose that processes at different levels of operation and explanation of at least a computational system may be occurring at the same time, and therefore should not be diagnosed as influencing each other causally. The relation between what is happening at the different levels of explanation is instead described as supervenient (or subvenient) (Rolls, 2021a).

Part of the aim of this paper is to make these proposed criteria for causality very explicit, in order to promote discussion of this approach to causality, as it may offer a useful way forward in helping to understand the relation between mental events and brain events, and for that matter between software events and hardware events in computers.

My answer to the first key aim of this paper is that the theory of causality should be extended to include the three criteria listed above, and to go beyond purely interventionist approaches to diagnosing causality, at least for systems such as the brain and the mind, and for conventional digital computers.

## Which Level of Explanation May Provide a More Accurate Account for the Cause of a Choice or Action: The Mental Level, or the Computational Neuroscience Level?

An appropriate level of description for the causes of events can be chosen in a levels of explanation account of the relation between the mind and the brain (Rolls, 2021a). Sometimes it may be the mental level, for example when we are explaining how we may have made progress with a problem such as the relation between the mind and the brain; and sometimes it may be the brain level, for example when we are considering which drug may be appropriate to treat a particular mental disorder.

However, it is interesting to consider at which level of explanation causality may be most accurate. It is well-known for example that confabulation can occur, and the rational mind may fabricate an account for why a choice was made or an action was performed. Part of the reason for confabulation by the rational system may be to help it maintain a long-term autobiographical narrative about the person’s self, and the need for the rational system to believe that it is in control, for otherwise it might stop trying (Rolls, 2012b).

**FIGURE 1:**
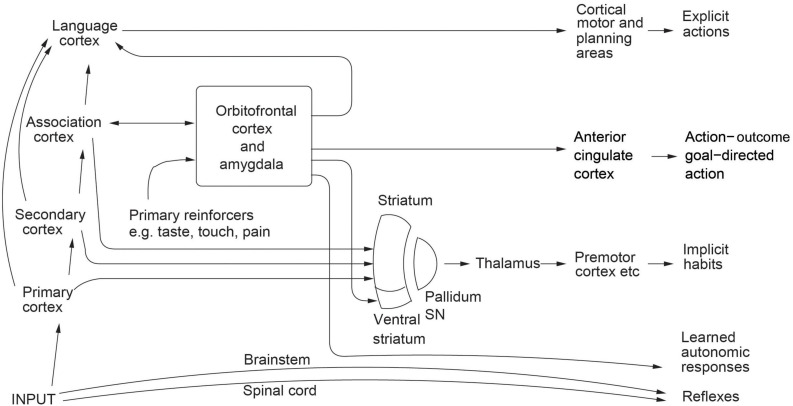
Multiple routes to the initiation of actions and responses to rewarding and punishing stimuli. The inputs from different sensory systems to brain structures such as the orbitofrontal cortex and amygdala allow the orbitofrontal cortex and amygdala to evaluate the reward- or punishment-related value of incoming stimuli, or of remembered stimuli. One type of route is *via* the language systems of the brain, which allow explicit (verbalizable) decisions involving multistep syntactic planning to be implemented. The other types of route may be implicit, and include the anterior cingulate cortex for action-outcome, goal-dependent, learning (Rolls, 2019); and the striatum and rest of the basal ganglia for stimulus-response habits (Rolls, 2014, 2021c). Pallidum / SN—the globus pallidus and substantia nigra. Outputs for autonomic responses can also be produced using outputs from the orbitofrontal cortex and anterior cingulate cortex (some of which are routed *via* the ventral, visceral, part of the anterior insular cortex) and amygdala (Rolls, 2021c). [From Rolls (2021c). Brain Computations: What and How. Oxford University Press: Oxford.] (9_4d.eps).

An example of confabulation is found in split brain humans who may say they prefer one house because it has some extras, or that there is no particular reason for their choice, when in fact they have been shown a picture to their non-dominant hemisphere that the other house is on fire (Gazzaniga and LeDoux, 1978; Gazzaniga et al., 2019). Confabulation may happen frequently when the emotional brain contributes an input to a decision, and the rational brain confabulates an explanation for why the choice was made, because there are multiple routes to action (Rolls, 2014; Figure 1). In such cases, we can know about the real cause of the decision or action only by knowing which brain systems were involved in taking the decision, and how the computation was performed that led to the decision, rather than by relying on any verbal explanation from the rational system that may be provided for the decision, for that might be a confabulation. For emotion-related decisions, it is suggested that confabulation by the rational system may occur frequently (Rolls, 2014). But when the decisions are taken by the rational system, it is more likely to be able to provide a correct causal account of the steps in the decision-making process, because the report comes from the same neural system involved in the reasoning (Rolls, 2020).

In patients with brain damage, confabulation is of course well known. It is common in patients with memory problems due to damage to the ventromedial prefrontal cortex (Schneider and Koenigs, 2017), or to the hippocampal memory system in for example Korsakoff’s psychosis associated with alcoholism (Dalla Barba and Kopelman, 2017). Although there are a number of different possible factors that account for confabulation in patients with brain damage (Dalla Barba and Kopelman, 2017), part of the problem may be a weaker signal in the memory system than is usual, so that the patient has to make up a rational explanation (in the form of a confabulation) in order to maintain a consistent model of the self (Rolls, 2020). This account may also fit why confabulation can occur in healthy people when the emotional decision-making system in the brain makes a decision, because the rational system has only imperfect access to the emotional decision system when the rational system is called on to provide reports. My hypothesis is that whether the emotional or the rational decision-making system actually takes a decision on a particular trial is itself a noisy decision-making process (Rolls and Deco, 2010; Rolls, 2011, 2016, 2020).

The overall implication of this consideration of “multiple routes to action” is that some levels of explanation may provide more accurate evidence about the causes of decisions and actions than others. The best way to understand the operation of a system may not necessarily be at the level at which a simple account can be provided and even verbally reported, in our example at the mental level. To understand the mind more accurately, and to be able to compare different types of mind, it may be important to know exactly what computations are being performed in the brain, as set out previously (Rolls, 2021a).

My answer to the second key question is thus that explanation of the causes of behavior and mental states at the mechanistic level of the operation of networks of neurons in the brain and what they are computing may provide a more accurate account for the cause of a choice or action than for example the report given by an individual at the mental level. Indeed, I argue that the best way of knowing about the properties of the system, including what it may be like to be the system, is to know exactly what computations are being performed in the system, rather than trying to make inferences about the system from tests such as the Turing test (Rolls, 2020, 2021a).

## The Question of Downward (Or Upward) Causation

It has been argued that downward causation may apply in some circumstances (Woodward, 2020, 2021a,b), but there is important discussion about this (Craver and Bechtel, 2007).

### Do Environmental Events Cause Changes in Gene Expression?

An example of possible downward causation that has been considered is that large scale environmental events may causally affect gene expression (Woodward, 2021a,b). But let us consider this further, in the way suggested in my within-levels of explanation approach to causality. If say an increase in environmental temperature led to genetic changes, this could occur in two main ways. One is that random genetic variation might lead to changes that might increase the size of the ears, or panting (both good for losing heat), and these might increase reproductive success for individuals who did not die from the heat. A second is that the gene expression for certain genes that for example promoted sweating might be turned on by their sensitivity (whether direct or indirect) to body temperature. In both cases, the causal account can be at the level of mechanistic biology, which provides a complete causal account of how a change in the environment might affect genes. Stating that the environment affects the genes in this case may be thought of as stating that whatever interventionist tests have been performed do not exclude that there is a relationship between the environment and the genes, but I argue that we can understand that there is causality when we analyze the steps involved at the lower mechanistic level, when the operation of causality becomes clear. Thus any account of this in terms of “downward causation” may just be referring to a state in which strong correlations may be present between levels, but with the causal mechanisms involved best described at a different level, of how it is at the biological level that changes in genes can be produced by for example temperature sensed within the individual.

### The Relation Between Neuronal Events and Mental States

Another example might be that excessive synaptic pruning or reductions in synaptic transmission produced by lower NMDA receptor efficacy may causally contribute to some of cognitive and behavioral symptoms of schizophrenia (Rolls, 2021b). As these changes in synaptic transmission relate to the symptoms (which involve the whole person), should this be considered as a case of across-level causation (Woodward, 2021a,b)? (In this case, it would be upward causation, from synapses to cognitive symptoms.) Examples of this type were considered by Rolls (2021a). The approach I take to such examples of relations between levels of explanation involving the brain, behavior, and mind is computational, that mental events can supervene on brain events, and that implies correlations between mental events and brain events, but that causality can best be understood as operating within a level of explanation. In the present case, the account is that reduced synaptic transmission (caused for example by high synaptic pruning or reduced NMDA receptor conductances) reduces the firing rates of populations of neurons, which destabilizes the attractor neuronal networks in the prefrontal cortex (Rolls, 2021b,c). Now these prefrontal cortex attractor networks are involved in maintaining items in short-term memory, and in holding on-line in short term memory the top-down bias required to bias processing in some parts of the brain thus providing a mechanism for top-down attention (Deco and Rolls, 2005a,b; Luo et al., 2013; Rolls, 2021c). The computational level of events in the brain thus provides a causal, computational, account of how these synaptic events alter behavior so that attention and short-term memory change. But the causal level is within-level in this approach, at the level of synapses, transmitters, receptors, and neuronal networks; and the behavioral changes occur at the same time, but are descriptions at a higher level of explanation. In such systems we can describe correlations between levels, or superveniences between levels of operation of the system, but the mechanistic, causal, computational account is best dealt with in this case at the brain level of explanation.

### The Relation Between Higher Level Laws and Lower Level Computations in the Brain

Another possible case considered as “downward causation” in physics is when a higher level Law “causes” an effect at a lower level (Ellis, 2020). Let us take as an example the interaction between neurons in a population that falls into a low energy attractor basin (Hopfield, 1982; Amit et al., 1985; Amit, 1989). This happens to be a system that is highly relevant to understanding the operation of the cerebral cortex, as the most characteristic attribute of cerebral cortex is the highly developed excitatory recurrent collateral local connections between pyramidal cells that enable local attractor networks to be implemented for short-term memory, long-term memory, top-down attention, decision-making etc. (Rolls, 2016, 2021c). If we have a set of non-linear neurons in a network with excitatory synapses of strength *w*_ij_ between each pair of neurons *i* and *j* and the firing rate of each neuron is *y*, and this forms an attractor network in which the synaptic weights reflect the stored memory patterns (see Rolls, 2016, 2021c), then the energy of the whole neuronal population can be expressed (Hopfield, 1982; Treves, 1991; Treves and Rolls, 1991) as

(1) $$\text{E}=-\frac{1}{2}\,\sum_{i,j}w_{i\,j}\,\left(y_{i}-<y>\right)\,\left(y_{j}-<y>\right)$$

where < *y* > is the average firing rate of all the neurons. This can be understood as follows. If two neurons *i* and *j* both have high firing rates (or in physics the magnetic spins are pointing in the same direction) and are connected by a strong synaptic weight, then they will support each other, and this will contribute to stability. If one neuron *i* has a high firing rate and *j* has a low firing rate and they are connected by a strong synaptic weight, then each neuron will tend to change the other into its state, and this will contribute to instability. In the same situation, if the linking weight is weak, this will make little contribution to the stability. The sum of all such interactions will be high when the system has reached stability as a result of interactions between the neurons, and this high stability can be expressed as a low energy *E* by using a - sign.

The interaction between the neurons (equivalent to spins in a physics model) can be analyzed at the population level (but not at the single neuron level) to show how the whole network can fall into an attractor state, and to show that the number of possible attractor states, for example the maximum number of different memory patterns, *p*_max_, that can be stored and correctly retrieved is approximately

(2) $${\text{p}}_{\text{max}}\approx \frac{C^{R\,C}}{a\,\text{ln}\,\left(1/a\right)}\,\text{k}$$

where *C*^RC^ is the number of recurrent collateral connections onto each neuron, *k* is a scaling factor that depends weakly on the detailed structure of the rate distribution, on the connectivity pattern, etc., but is roughly in the order of 0.2-0.3 (Treves, 1991; Treves and Rolls, 1991), and *a* is the sparseness of the representation. [For binary neurons with either a high or a zero firing rate, the sparseness is the proportion of neurons with a high firing rate (Treves and Rolls, 1991; Rolls, 2021c)]. For example, for *C*^RC^ = 12,000 associatively modifiable recurrent collateral synapses onto each neuron, and *a* = 0.02, *p*_max_ is calculated to be approximately 36,000.

One concept of causality that has been advanced for systems with different levels is that because a Law can be specified for a system such as what is shown in Eqn (2) at a high level (the population of neurons level), then that Law or rule of operation formulated at the high level provides “downward causation” to the lower level, to in this case result in the number of stable attractor basins being limited to what is shown in Eqn. (2) (Ellis, 2020).

But that is not how I see the system as operating in terms of causality. The individual neurons at the lower level do not wait for a top-down signal from the population level to tell them what to do next. Instead, it just is a property of the whole system that the individual neurons at the lower level operate as neurons each with a certain number of connections to the other neurons, and the result of the lower level interactions between the neurons is that only a certain number of stable states can be stored and correctly retrieved. To elucidate further, when we simulate such an attractor network in a computer, we set up for example neurons with threshold linear activation functions, and modify the synaptic connections between the neurons to store the memory patterns, and then we let the system run (Rolls, 2012a,2021c). We find that as we increase the number of memory patterns stored in the system, at some point, the critical capacity, the recalled memories become very poor, and the system no longer works as a memory system (Rolls, 2012a). But we do not include in the program that we write that the neuron-level implementation should check up to some higher level to find out if the number of patterns specified by the Law specifying the critical capacity has been exceeded, and if so to fall into a random neuronal firing (or spin) state. Nor is there a high-level part of the program that knows about Eqn (2) and checks if *p* is too high, and if so causes the lower level to fall into a random spin state (i.e., random set of neurons firing). So the operation of the system is implemented only at the lower level, and that is where causality acts, by the firing of individual neurons influencing other neurons through the modified synaptic weights. Now of course the operation of the system in terms of its storage capacity can be explained, and analyzed, at the higher level, where the interactions between the whole population of neurons can be understood, and specified as rules or Laws of the operation of the system. But that does not mean that the higher level rules or Laws that describe the operation of the whole system have to act down to the lower level to cause effects there at the low level, whether synchronously, or after a time delay. Thus I reject the concept (Ellis, 2020), at least in relation to the operation of the brain, that Laws that apply at a high level act by “downward causation” to control the operation of the system at a lower level. The high level Laws just express some properties of the system.

### “Downward Causation,” Confabulation, and Correlation

An implication of the treatment above of confabulation at an upper level of the system is rather relevant to the issue of possible downward causation. We should be wary (due to the possibility of confabulation), because a claimed example of downward causation may in fact be incorrect, for in the case of confabulation the mental thought that is expressed is not in fact in the causal chain at all of why a behavior or action may have occurred. Indeed, many examples of what might be claimed to be top-down causation may be because the concept at the high level is inadequately defined for it to be really testable as a cause. Take the example that the position in the status hierarchy might be considered to be the cause for altered gene expression which alters serotonin levels. Should we consider this to be a case of “downward causation,” as suggested (Woodward, 2021b)? This is likely to reflect a general association or correlation. Position in a dominance hierarchy is likely to reflect the outcome of agonistic interactions such as fights, and we know that there is considerable individual variation in sensitivity of the lateral orbitofrontal cortex, which decodes this non-reward, to not winning or losing (Rolls et al., 2020; Xie et al., 2021). Moreover, the non-reward might lead to active behavior, perhaps initiating a fight, or to passive behavior, to opt out of trying (Rolls, 2014). Which of these behaviors is chosen depends on impulsiveness, which is influenced by similar brain regions (Dalley and Robbins, 2017). And what happens to serotonin system gene expression is likely to depend causally on the exact chain of processing and computations, and can be understood at that level. So a putatively causal statement that “status hierarchy causes gene expression changes” (Woodward, 2021b) may reflect a general correlation, but there is no necessary relation, and this is not a very substantive form of causality. The attempt at a top-down causal explanation here seems to reflect instead a general correlation; and the causal factors involved can be described at the more mechanistic neural level, of the extent to which the lateral orbitofrontal cortex non-reward neurons are activated in an individual by losing or not winning (Thorpe et al., 1983; O’Doherty et al., 2001; Rolls et al., 2020; Xie et al., 2021), and by the personality of the individual such as impulsivity and sensitivity to punishment, which do at the neural systems level provide an account of the causal links in the chain that lead to how gene expression might be altered.

### What Defines a Level of Operation/Explanation in the Brain and Mental Systems? A Computational Neuroscience Approach

It is useful to provide some guidance on what defines a level of operation / explanation, at least for what is being considered here, neural and mental systems. Different levels can be defined by for example matters of scale and numbers. Some examples follow.

One level is the neuron level. There are very many small ion channels in a neuron that together with their arrangement on a neuron influence whether the neuron will generate an action potential. Each neuron has one output stream of information, reflected by its action potentials, directed to perhaps 20,000 other neurons. Each neuron has perhaps 20,000 synaptic inputs from other neurons, which act on the ion channels to influence whether a neuron produces an action potential. I argue that this neuron-level is one computational level of operation of the system, for what the neuron computes is reflected in its single output stream of information, its action potentials transmitted to 20,000 other neurons. This is the type of single neuron computational level of understanding that can be commonly applied in the mammalian brain (Rolls, 2021c). I include in this level the fact that it is a property of some ion channels that the currents that they pass depend on the voltage across the membrane, as for the n-methyl d-aspartate receptor (NMDAr) which is important in learning (Rolls, 2021c). I also include in this level that for the synaptic strengths to modify and be retained during learning, genes may need to be activated to help produce the chemicals needed to alter the structure and strength of the synapse (Kandel, 2001). It is essential to understand the operation at this level, in terms of the information conveyed by the train of action potentials from a single neuron, which can be 0.3 bits in even a short time period of 20–50 ms (Tovee and Rolls, 1995; Rolls et al., 1997b, 1999), but which is largely independent from even nearby neurons (up to tens of neurons), as shown by the evidence that the information rises linearly with the number of single neurons being recorded (Rolls et al., 1997a; Rolls and Treves, 2011; Rolls, 2021c).

A higher computational level is that of a population of neurons. There are very many neurons in a population that influence how and what the population computes, with one example being the type of attractor network described above. In this, as shown in equation 1, coalitions of neurons linked by strong synapses and high firing rates can be formed and form a stable basin of attraction, and have the “emergent” property of completion of the whole memory from any part (Hopfield, 1982; Rolls, 2021c). These networks are typically localized to a small area of neocortex, to minimize the axonal connection length between the neurons that must interact in the same network. Typically there will be 100,000 excitatory neurons in such a local network, given approximately 10,000 synapses per neuron devoted to recurrent collateral connections, and a dilution of connectivity of about 0.1 (Rolls, 2021c). Other types of network include pattern association networks, and unsupervised competitive networks to learn new representations (Rolls, 2021c). In all cases, the computation can be understood at the network level, and not at the single neuron level (Rolls, 2021c). There is a characteristic time-scale of operation here too, in the order of 10–15 ms even for an attractor network, and determined primarily by the time constant of the excitatory AMPA (α-amino-3-hydroxy-5-methyl-4-isoxazolepropionic acid) receptors that connect the excitatory neurons (Battaglia and Treves, 1998; Panzeri et al., 2001; Rolls, 2021c). These dynamics are made fast because the integrate-and-fire neurons have a low spontaneous firing rate, so that some neurons are always very close to threshold before the stimulus is applied, and start exchanging information through the trained synapses very rapidly. The dynamics of the operation of the system, while it falls into its attractor state, which is one of a limited number of possible stable memory states, occurs continuously in time, and does not require the neurons to ask the next level up, at which the theory of the number of stable states can be analyzed (Hopfield, 1982; Treves, 1991; Treves and Rolls, 1991), whether the current stable state meets the criteria: the neuronal population just falls into one of its possible stable states based on interactions between the population of neurons. The fact that transmitters such as acetylcholine with widespread effects modulate the excitability of the whole population of neurons of course influences how stable the states are (Rolls and Deco, 2015b), but does not raise new issues about causality.

Another level of operation is that involved in solving a problem such as proving Pythagoras’ theorem, or writing a paragraph of text. This is a typically serial computational operation that may require many populations of neurons (of the type just described) exchanging information with each other with different steps to the argument, which together may take seconds or minutes, not the 10–15 ms for a single network to operate. Another example is the production of speech, which is a serial operation, and which might be implemented by a forward trajectory through a state space of different attractor networks each representing a different part of speech (e.g., subject, verb, and object), and each attractor network connected with stronger forward than backward connections to the next network (Rolls and Deco, 2015a). Thus the spatial scale here is different, with many populations of neurons involved; and the timescale is different, with serial operations being performed. Due to the almost random spiking times for a given mean firing rate of individual neurons, the population of neurons under this stochastic influence, may sometimes jump to a new location in the high-dimensional space, and this is likely to be important in creativity (Rolls and Deco, 2010; Rolls et al., 2010; Liu et al., 2018; Sun et al., 2019; Rolls, 2021c). At this level of explanation, we can see how sets of networks could implement a multistep algorithm.

At a higher level of explanation, we might specify the operation at an algorithmic level, for example the computational steps taken to prove Pythagoras’ theorem, or the steps in the firing cycle of a combustion engine, or the stages in the life history of a dragon fly. This is the most useful level for analysis of whether the algorithm operates correctly, and to describe the algorithm to other individuals. And causality can be understood at this level, as progress with one step of the algorithm can enable the next step to occur. But the processes can also be understood as operating at the lower level of sets of neuronal networks in the brain, which reflect in their connections and operation what has been learned previously by interaction with the environment, and so can be constrained by what has been learned to implement the steps of the algorithm with causality operating at that level of sets of neuronal populations, with the learned constraints influencing what is computed without the need for top-down causality of what can be explained at a higher level to cause things to change, after a small delay, at the lower level of sets of populations of neurons.

At a higher level of explanation and operation of the system, it might be that when the neuronal networks are performing a particular type of computation, perhaps monitoring a multi-step chain of reasoning using higher order syntactic thoughts grounded in the world, that it is a property of such a system that it feels like something to be having those higher order thoughts about oneself that are grounded in the world. That is the computational processor that I suggest becomes engaged when we report phenomenal consciousness. Part of the argument is that much global processing can take place without phenomenal consciousness, for example riding a bicycle for a time while thinking about something else (such as a theory of consciousness), so that a special type of computation appears to be involved when we have phenomenal feelings of consciousness (Rolls, 2004, 2008, 2011, 2020).

So scale and number seem often to be useful in describing levels. They provide an independent way of defining a level to ideas of for example whether any one scale (or several scales) is complete (Ellis, 2020). For me, no one scale or level of explanation or operation suffices for a complete explanation, in that although causality operates within a level, understanding of how the system operates at different levels of operation may be useful. For example, understanding at the neuronal / pharmacological level may be useful for treatment, whereas understanding at the level of reasoning may be useful to understand Pythagoras’ theorem. I consider that the whole world is a set of different levels of both operation and explanation, and they are linked by the ideas of supervenience and subvenience, or better convenience (see below), which are non-causal but different properties of the operation of the same system, understood and analyzed at different levels, with causality operating within each level, and not between levels. A consequence of my approach is that causality can be described as operating simultaneously as each of several levels of operation or explanation, but this does not imply multiple causes: the operations at each level provide different ways of describing and analyzing the computational properties of what is a single system.

### Summary

My response to the third key issue, possible cases of “downward causation” (from a higher level to a lower level), is that they can be accounted for, at least for mental vs brain levels of operation and explanation, by the approach to causality described here, in which causality operates within but not between levels. Moreover, the neural level is more substantive, for it enables the links in the causal chains that might lead to different effects to be followed across time, whereas events expressed in words at a higher mental level may be too imprecise to reflect more than correlations; and further, may reflect confabulation.

## Implications for Dualism and Physical Reductionism

Descartes took a dualist approach to the relation between the mind and the brain (Descartes, 1644), and that raised the problem of how the mind and brain relate to each other, which has been a problem in the philosophy of mind ever since. One solution has been to propose a reductive physicalism, in which it is argued that mental events can be reduced to brain events, with no differences in kind (see Kim, 2011; Carruthers, 2019).

**FIGURE 2:**
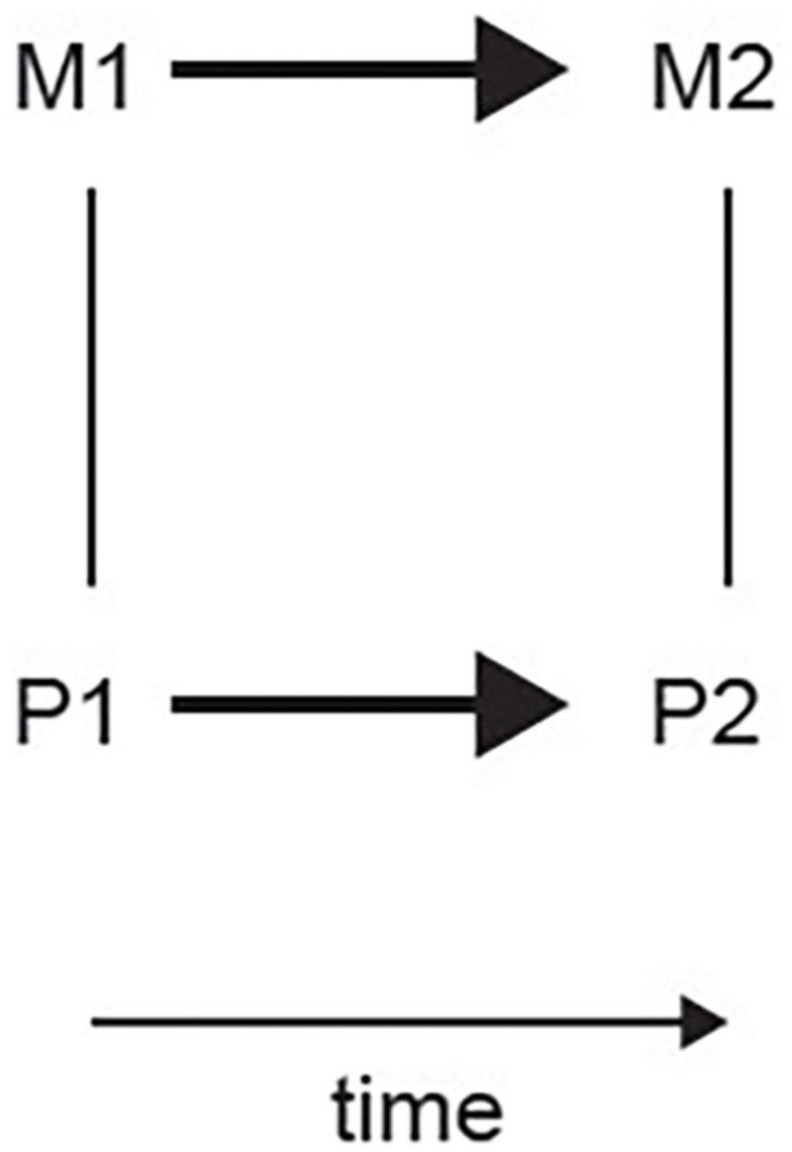
Schematic representation of the relation between physical brain states (P1 and P2) and mental states (M1 and M2). Undirected edges indicate supervenience / subvenience relations which apply upward and downward and are non-causal. The edges with an arrow indicate a causal relation (supervenience.eps).

The approach that I have proposed is that the mental events [including phenomenal consciousness (Rolls, 2020)] can be different in kind from brain events, and that the mental events supervene computationally on brain events. How the computational levels relate to each other has been described with examples by Rolls (2020, 2021a). My approach proposes that there is a necessary relation between a lower level and an upper level of explanation / operation, with events at the neural level always (i.e., necessarily) being related to some mental event at the higher level; and vice versa. The correlation between the appropriate events at the neural level and at the mental level will be high. But this relation between the lower level and the higher level is not causal, because the events at the lower (neural) and higher (mental) level happen at the same time (Figure 2). Some philosophers use the term “supervenience” for how the high level relates to the lower level. However, the term “supervenience” may carry with it some implications for some philosophers. In this context, another term that I suggest for this is “convenience,” which from the Latin means “coming together” (con-veniens). This term, “convenience,” has the advantage that it could be applied to both supervenience and subvenience, and does not carry with it the implications of the term “supervenience” as it may be understood by some philosophers. My proposal is that in at least a computational system such as the brain, the higher level, for example mental, events are what are implemented by the lower level, neural, events, but that this is not a causal relationship because the events at the different levels happen at the same time, and is a “convenient” relationship. This computational approach to the relation between mental and brain events may offer a solution to the problems of dualism and of reductive physicalism, with the relations summarized in Figure 2.

Given this computational approach to the relation between the brain and the mind, the events at the mental level can be different in kind at the mental level from the neural level. The mental events might include having thoughts about one’s own syntactic thoughts, in order to correct one’s lower order multi-step planning and reasoning. If the reasoning and planning is grounded in the world, if it is for example about rewards and punishers that might have implications for life or death of the individual who can think ahead about its own future, then I suggest that one of the properties of the system may be phenomenal consciousness (Rolls, 2004, Rolls, 2007a, Rolls, 2007b, Rolls, 2008, Rolls, 2011, Rolls, 2012b, Rolls, 2020). The thoughts at that mental level are an example of what I mean by differences in kind from a lower level of explanation, which in this case might be the level of the operation of neurons in the brain, or of populations of neurons to implement a particular computation. All of those firings and the closely related network operations (Rolls, 2021c) are different I suggest in kind from mental events including that we feel conscious (Rolls, 2020).

## Summary and Conclusion

In order to understand the relation between the mind and the brain, and whether mental events cause brain events, or vice versa, it is important to have a theory of causality that is useful in computational neuroscience. Here I have proposed an approach to causality at least within computational neuroscience that goes beyond interventionist tests to include also temporal order, and that the causality should operate within levels of operation or explanation, and not between levels.

Second, I have shown that although different levels of explanation for the operation of the system may be useful for different purposes, some levels of explanation may be more accurate than others. In particular, I propose that the mechanistic neural level may be more accurate and reliable than the mental level provided by verbal report of the causes for actions, because for example of confabulation which can occur given that the brain contains multiple routes to produce behavior. It is in principle possible to know which of the multiple routes to action illustrated in Figure 1 was engaged for some behavior or decision, by measuring which system in the brain is active on a particular occasion (McClure et al., 2004; Rolls, 2021c).

Further, I propose that the best way of knowing about the properties of the system, including what it may be like to be the system, is to know exactly what computations are being performed in the system, rather than trying to make inferences about the system from tests such as the Turing test. Third, I argue that the possible cases of “downward causation” (from a higher level to a lower level) that are discussed in the literature can be accounted for by the approach to causality described here, in which causality operates within but not between levels.

Overall, these proposals offer a computational neuroscience-based approach to the problems raised by both dualism and reductive physicalism; and an approach to understanding causality in computational systems.

## Data Availability Statement

The original contributions presented in the study are included in the article/supplementary material, further inquiries can be directed to the corresponding author/s.

## Author Contributions

The author confirms being the sole contributor of this work and has approved it for publication.

## Conflict of Interest

The author declares that the research was conducted in the absence of any commercial or financial relationships that could be construed as a potential conflict of interest.
